# Influence of *Mikania laevigata* Extract over the Genotoxicity Induced by Alkylating Agents

**DOI:** 10.1155/2013/521432

**Published:** 2013-03-05

**Authors:** Daliane Medeiros Mazzorana, Vanessa Nicolau, Jeverson Moreira, Patrícia de Aguiar Amaral, Vanessa Moraes de Andrade

**Affiliations:** ^1^Laboratório de Biologia Celular e Molecular, Programa de Pós-Graduação em Ciências da Saúde, Universidade do Extremo Sul Catarinense (UNESC), Avenida Universitária 1105, Bairro Universitário, 88806-000 Criciúma, SC, Brazil; ^2^Grupo de Estudos Etnofarmacológicos Visando à Obtenção de Substâncias Bioativas, Universidade do Extremo Sul Catarinense (UNESC), Avenida Universitária 1105, Bairro Universitário, 88806-000 Criciúma, SC, Brazil

## Abstract

Medicinal plants are still widely used worldwide; yet for some species, little or no information is available concerning their biological activity, specially their genotoxic and antimutagenic potential. *Mikania laevigata* (Asteraceae) is a native plant from South America, and its extracts are largely used to treat respiratory complaints. The aim of the present work was then to evaluate, *in vivo*, the potential biological activity of *M. laevigata* on the genotoxicity induced by methyl methanesulfonate (MMS) and cyclophosphamide (CP), using the comet assay. Male CF1 mice were divided into groups of 5-6 animals, received by gavage 0.1 mL/10 g body wt of water, *Mikania laevigata* extract (MLE), MMS, and CP. Results showed that treatment with 200 mg/kg of the MLE previously to MMS and CP administration, respectively, reduced the damage index (DI) in 52% and 60%, when compared to DI at 24 h. Pretreatment also reduced the damage frequency (DF) in 56% (MMS) and 58% (CP), compared to DF at 24 h. MLE administration has been shown to protect mouse DNA from damage induced by alkylating agents; this corroborates to the biological activities of *M. laevigata* and points towards the need of plant compounds isolation to proceed with further studies.

## 1. Introduction

 The diversity of plant species in Brazil is a potential source of biologically active compounds. The use of medicinal plants is a generalized practice in folk medicine for the treatment of different types of diseases [1–4], and they are promising sources for the discovery of novel potentially therapeutic agents [5]. However, in the majority of cases, there is no proof of the efficacy of treatment popular use, nor there has been an adequate evaluation of medicinal plants for possible adverse effects [6].

 Among various medicinal plants used in Brazil stands out *Mikania* genus plant (Asteraceae family), a subscrub creeper of woody branches, known popularly as “guaco” [7]. The genus *Mikania* includes around 450 species, many of which are found in Brazil and other South American countries, besides tropical regions of Asia and Africa [8]. Although the species considered in the Farmacopeia Brasileira is the *Mikania glomerata* (in the use of syrup), the *Mikania laevigata* is the common one commercialized due to similarities to both internal and external morphology, majority of chemical substances (coumarin), and the same habitat [9].

*Mikania laevigata* has been widely used as infusions or plasters, while the crude extract of this species is commonly commercialized as phytomedicine, mainly to treat inflammatory disorders, such as bronchitis, chronic lung diseases, and bronchial asthma [10]. Among other effects that have been described to the *Mikania laevigata* are anti-inflammatory, antioedematogenic [11], antiulcerogenic [12], antimicrobial [13, 14], antispasmodic, and bronchodilatory [15].

 Previous studies have described the presence of various chemical constituents of *Mikania laevigata*, including coumarin, kaurenoic acid, stigmasterol, cinamoil grandiflorus acid, and dihydrocoumarin [16]. Coumarin is a major chemical substance found in *Mikania laevigata*, and there is a relationship between it and the pharmacological action of this plant. For this reason, it has been used as a chemical marker in pharmaceutical presentations [17, 18]. Furthermore, studies have pointed coumarin as a potential substance for the treatment of cancer [19], with growth inhibition activity and cell kill in various tumor cells lines [20], and a liver toxicant and not showing toxicity in the reproductive system in male Wistar rats [21]. 

 Many studies have been developed to demonstrate the actions of *Mikania*, expanding further our knowledge about this plant. Suyenaga et al. [11] have developed a study with *Mikania laevigata*, in which results indicated that this plant was more effective in producing the anti-inflammatory activity than *Mikania glomerata*. There is little information in the literature on the action of *Mikania laevigata* in genetic level, but Simmons and Snustad [22], using *Mikania glomerata*, reported that infusion of this species could cause damage that was not reversible by the cell's DNA repair system. Costa et al. [23] also noted an increase in genotoxicity, using infusion of *Mikania glomerata* in highest doses.

 While there is widespread use of medicinal plants in popular medicine for the treatment of different diseases, not all species are harmless to human health and may present toxic and mutagenic substances in their phytochemical composition [4]. On the other hand, a protective action of phytochemical compounds on genetic material has been reported, leading to its repair or to preserving its integrity [24]. Considering that there are few data on the biological effects of the extracts of *Mikania laevigata*, mainly in genetic level, this work aims to evaluate, *in vivo*, the effect of *M. laevigata* on the genotoxicity of the alkylating agents Methyl methanesulfonate (MMS), a direct acting mutagen, and cyclophosphamide (CP), that requires metabolism to become active, using the comet assay.

## 2. Materials and Methods

### 2.1. Identification and Extraction of Plant Material

Aerial parts (leaves) of *M. laevigata*, family Asteraceae, were collected in Grão Pará, SC, Brazil in September 2008.

A voucher specimen (no. CRI 7379) of *M. laevigata* was deposited at Herbarium Pe. Dr. Raulino Reitz, Universidade do Extremo Sul Catarinense, Criciúma, SC, Brazil. The leaves were allowed to dry under air circulation (40°C) for 3 days and were powdered. The resulting powder (400 g) was subjected to dynamic maceration with 2 L of ethanol : water (70 : 30, vol/vol) solution for 3 hours. The extract was filtered, and this procedure was repeated twice. The solvent was evaporated in a rotary evaporator, and the residue was dissolved in distilled water [14, 25]. 

### 2.2. Phytochemical Screening

The phytochemical analysis (flavonoids, tannins, anthraquinones, alkaloids, saponins, coumarins, and cardiac glycosides) of the aerial parts of *M. laevigata* was carried out according to the methods described by Harborne [26]. The thin layer chromatography analyses were performed following systems and developers indicated by Wagner and Bladt [27], and aluminum chloride colorimetric method was used for flavonoids quantitative determination [28]. Each plant extracts (0.5 mL of 1 : 10 g) in methanol were separately mixed with 1.5 mL of methanol, 0.5 mL of 2% aluminum chloride, 0.1 mL of 1 M potassium acetate, and 2.8 mL of distilled water. It remained at room temperature for 30 min; the absorbance of the reaction mixture was measured at 425 nm with Biospectro Model SP-22 UV/Visible spectrophotometer.

### 2.3. Animals

Male CF1 mice between 6–8 weeks and weighing between 30 and 50 g were obtained from the Central Animal House of the Universidade do Extremo Sul Catarinense and caged in groups of five with six animals each, provided with commercial mice chow and water *ad libitum*, and maintained on a 12-hour light : 12-hour dark cycle. All studies were performed in accordance with National Institutes of Health guidelines and with the approval of the Ethics Committee of the Universidade do Extremo Sul Catarinense. 

### 2.4. Treatments and Test Substances

The treatment groups received by gavage 0.1 mL/10 g body wt of (a) water, (b) *Mikania laevigata* extract (MLE), (c) MMS, and (d) CP (Table 1). Dose levels of MMS were 40 mg/kg body/wt and 25 mg/kg body/wt for CP. All substances were prepared just before treatment and protected from light. 

### 2.5. Blood Sample Collection

One or two drops of blood were collected from mouse tail tips by means of a small incision [29]. Animals were sampled 24 and/or 48 h after treatment (Table 1). Drug administration and blood sampling were performed as described previously [30]. Peripheral white blood cells are among the most used cells for genotoxicity studies, mainly with the comet assay. They circulate through the entire body and are easily obtained.

### 2.6. Comet Assay

The alkaline comet assay was performed as described by Singh et al. [31] with adaptations by da Silva et al. [32]. Blood samples were drawn from the caudal vein of each mice and mixed with the anticoagulant heparin.

Cells isolated from tissues (5–10 *μ*L) were embedded in a layer consisting of 95 *μ*L of 0.75% low melting point agarose gel on frosted slides and immersed in a lysis buffer (2.5 M NaCl, 100 mM EDTA, and 10 mM Tris [pH 10.0–10.5] with freshly added 1% Triton X-100 and 10% dimethyl sulfoxide) for a minimum of 1 hour and a maximum of 1 week. Subsequently, the slides were incubated in freshly made alkaline buffer (300 mM NaOH and 1 mM EDTA, pH > 13) for 20 minutes. The nuclei were electrophoresed for 20 minutes at 25 V (0.90 V/cm) and 300 mA, and then the alkali was neutralized with 0.4 M Tris (pH 7.5). After the neutralization, the slides were fixed (15% w/v trichloroacetic acid, 5% w/v zinc sulfate, and 5% glycerol), washed in distilled water, and overnight dried. The gels were rehydrated for 5 min in distilled water and then stained for 15 min (37°C) with a solution containing the following sequence: 34 mL of Solution B (0.2% w/v ammonium nitrate, 0.2% w/v silver nitrate, 0.5% w/v tungstosilicic acid, 0.15% v/v formaldehyde, and 5% w/v sodium carbonate) and 66 mL of Solution A (5% sodium carbonate). The staining was stopped with 1% acetic acid and the gels were air-dried [33].

### 2.7. Microscopic Analyses

**Figure 1:**
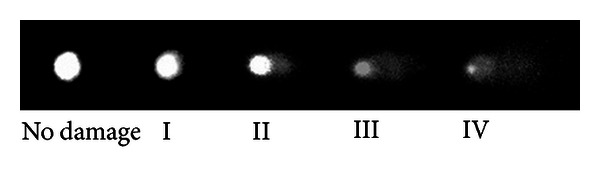
Comet assay. Evaluation of DNA damage using ethidium bromide (400x). The cells are assessed visually and received scores from 0 (undamaged) to 4 (maximally damaged), according to the size and shape of the tail.

Cells were scored from 0 (undamaged) to 4 (maximally damaged) according to the tail intensity (size and shape), resulting in a single DNA damage score (damage index) for each sample and, consequently, for each group (Figure 1). Thus, a damage index (DI) of the group could range from 0 (completely undamaged—100 cells × 0) to 400 (maximum damage—100 cells × 4) [34]. The percentage damage frequency (DF) was calculated for each sample on the basis of the number of cells with a tail versus with no tail.

### 2.8. Statistical Analysis

Student's *t*-test was used to compare DNA damage values between the different times (24 h versus 48 h, 24 h versus MLE pretreatment, and 48 h versus MLE posttreatment). Analysis of variance (ANOVA) was used to compare DNA damage induced by different substances at the same time (24 or 48 h). A parametric ANOVA was used when data showed normal distribution and were homogeneous in variance. In this case, the Tukey *post hoc* test was applied for multiple comparisons. Statistical significance was considered at a level of *P* < 0.05. All statistical analyses were performed independently for the two parameters evaluated. The statistical package used was BioEstat 5.0.

## 3. Results

After exposure of mice to water and MLE, little damage was seen in mouse peripheral blood cells sampled at 24 h through the comet assay (Table 2). There was no difference between these compounds for both the damage index as for the damage frequency. A slight increase in DNA damage was observed in these groups at 48 h of exposure, with significance for both groups compared to 24 h (*P* < 0.001, *t*-test); however the extent of DNA damage did not differ between mice treated with water and MLE (Table 2).

 At 24 h of exposure, MMS and CP were genotoxic according to both parameters evaluated in comparison with water and MLE treatments (*P* < 0.01, ANOVA, Tukey) (Table 2). A reduction in DNA damage was observed for both MMS and CP at 48 h, being significant in relation to 24 h in both groups (*P* < 0.001, *t*-test). Although decreasing in relation to 24 h values, the DNA damage levels for MMS and CP at 48 h remained significantly higher in relation to water and MLE (for DI and DF, *P* < 0.01, ANOVA, Tukey) treatments (Table 2).

When the level of DNA damage in blood cells of mice treated with MMS and CP and sampled at 24 h was compared with mice pretreated with MLE, this compound was able to induce significant reduction in DNA damage caused by alkylating agents in both evaluated comet assay parameters (*P* < 0.001, *t*-test) (Table 2). 

Posttreatment with MLE induced significant reduction in DNA damage in both parameters in blood cells for both mutagens in mice sampled at 48 h (*P* < 0.001, *t*-test) (Table 2).

The phytochemical analyses of *M. laevigata* extract indicated the presence of coumarins, flavonoids, alkaloids, cardiac heterosides, and tannins. Other secondary metabolites such as anthraquinones and saponins were not detected.

## 4. Discussion

Medicinal plants and their derivatives have been used as an alternative to synthetic medicines in many countries. The major compound found in *Mikania laevigata* is coumarin, which has been described as being responsible for the pharmacological actions and as a chemical marker of this plant [17, 18].

Assessment of antigenotoxic potential of *Mikania laevigata* extract (MLE) is very important, whereas antimutagenic substances present in some plants have been shown to help in the prevention of cancer and other diseases [35–38]. 

In our experiments, low levels of DNA damage in blood cells of mice treated with different concentrations of extract and infusion of *Mikania laevigata* by the comet assay were detected. The damage index was similar to the groups treated with water and with a significant low value in relation to the groups treated with the mutagens MMS and CP that have demonstrated high levels of DNA damage. These results demonstrate that the *Mikania laevigata* is not genotoxic to cells and also proved the efficiency of the assay. In a previous study, there was also an evidence that the extract of this plant showed no signs of mutagenicity in the *Salmonella*/microsome assay (Ames assay) [39]. Additionally, da Silveira e Sá et al. [40] working with Wistar rats showed that administration of *Mikania glomerata* extract to these animals was not able to change any of the parameters studied, suggesting absence of mutagenic effect. However, although the studies of Fernandes and Vargas [39], da Silveira e Sá et al. [40], and ours have not demonstrated genotoxic activity of this plant, Martins and Santos [41], have reported that both *Mikania glomerata* and *Mikania laevigata* showed a toxic effect only following excessive consumption. 

Oral administration of MLE in mice showed a reduction in the frequency and index rates of DNA damage induced by antineoplastic agents used in this study. With regard to cyclophosphamide (CP), the reduction was 60% and 65% for the DI in the groups pre- and posttreated, respectively. In relation to DF, the reduction was 58% and 54% in the groups pre- and posttreated, respectively.

With respect to DNA damage caused by the alkylant agent MMS, the reduction of the DI was 52% and 33.5% in the groups pre- and posttreated, respectively. In relation to DF, the reduction was 57% and 20% in the groups pre- and posttreated, respectively. These results show that the hydroalcoholic extract of *Mikania laevigata* has a protective and reparative effect and could be considered not only in relation to its multiple pharmacological properties but also to reduce the genotoxicity of chemotherapeutic agents on normal cells.

Many authors attribute the anti-inflammatory and healing properties of MLE to the presence of coumarin, flavonoids, and tannins, and several studies have been carried out to verify this contribution [7, 42]. Our phytochemical screening of MLE showed the presence of flavonoids, phenolic compounds, tannins, coumarin, and alkaloids in accordance with the previous results. 

The therapeutic use of plant containing flavonoids is vast, and many cases are still empirical. Although some results have shown that flavonoids may have a mutagenic effect, in general, these compounds are considered beneficial. Other research suggests that some flavonoids are responsible for antitumoral action. Used in the preventive chemotherapy of cancer, they showed the ability to interact on the genesis of cancer, blocking the stage of promotion, by inhibiting the synthesis of ornithine decarboxylase [43].

MMS can methylate nucleophilic regions of DNA and amino acid molecules, particularly at nitrogen atoms. MMS's genotoxicity is mediated by base modifications, which weaken the N-glycosylic bond, leading to depurination/depyrimidination of DNA strands and the appearance of alkali-labile abasic sites (AP sites). The removal of AP sites by AP endonucleases cleaves DNA adjacent to these sites and generates DNA strand breaks in DNA [44, 45]. Pretreatment and posttreatment with MLE reduced MMS's genotoxicity about 52% and 33.5% in DI (57% and 20% in DF), respectively. Thus, MLE was both preventive and reparative for the damage caused by MMS. In pretreatment, phenolic compounds could have competed as target site for alkylation. With respect to posttreatment, both phenolic compounds could have influenced the kinetics of repair [46].

CP is absorbed well after oral administration. The parent compound is widely distributed throughout the body with a low degree of plasma protein binding (20%). The half-life of CP is between 6 and 9 hours [47]. Once activated, CP can, besides monoadducts, also induce the formation of DNA-DNA and DNA-protein crosslinks [48]. CP has the ability to generate free radicals that cause endothelial and epithelial cell damage [47].

Pretreatment with MLE slightly reduced the level of DNA damage induced by CP (60% and 58% reduction in DI and DF, resp.), while MLE posttreatment induced a significantly higher reduction in DNA damage (65% and 54% reduction in DI and DF, resp.). Since CP requires metabolic activation before inducing DNA damage, it is likely that “guaco” components, such as phenolic compounds, alter the rate of metabolization and/or detoxification. In posttreatment, damage reduction was higher because both compounds could act as reactive species quenchers and DNA repair pathways modulators. Moreover, phenolics could have stimulated phase II enzymes and eliminated CP metabolites [29]. It is important to consider the kind of DNA damage generated by CP, particularly crosslinks. Such lesions can retard the migration of DNA fragments and lead to a wrong evaluation of the extent of DNA damage [48, 49]. 

### 4.1. Conclusions

In conclusion, consumption of MLE can be both protective and reparative of DNA damage induced in mouse blood cells by alkylating agents. Such protective effects of MLE differ depending on the mode of action of the mutagen. Our results demonstrate the ability of the *in vivo* comet assay to detect *in vivo* modulation of MMS and CP genotoxicity by MLE. 

Further studies are recommended to determine the conditions for the use of this plant *in vivo* that would offer the benefits of their therapeutic properties without putting at risk the human health.

## Figures and Tables

**Table 1:** Experimental procedures.

Procedure		Exposure schedule	
	0 h	24 h	48 h
Control	1st water treatment	1st blood sampling	
		2nd water treatment	2nd blood sampling
	1st extracts treatment	1st blood sampling	
		2nd extracts treatment	2nd blood sampling
Pretreatment	1st Treatment (extracts)	1st blood sampling	
		2nd treatment:	2nd blood sampling
		(1) MMS (2) CP	
Posttreatment	1st treatment (agents)	1st blood sampling	
	(1) MMS (2) CP	2nd treatment (Extracts)	2nd blood sampling
Alkylating agents	Agentstreatment: (1) MMS (2) CP	1st blood sampling	2nd blood sampling

**Table 2:** Detection of DNA damage by comet assay (DF and DI) in blood cells of mice exposed to water, *Mikania laevigata* extract (MLE), and/or cyclophosphamide (CP) or methyl methanesulfonate (MMS) and sampled at 24 h (with and without pretreatment with MLE) or 48 h (with and without posttreatment with MLE).

Substances	Single doses (mg/Kg)	Schedule and comet assay parameters			
		24 h	48 h	Pretreatment with MLE extract^a^	Posttreatment with MLE extract^b^
		Damage index			
		DI ± SD	DI ± SD	DI ± SD	DI ± SD

Water (*n* = 6)	—	2.3 ± 1.5	8.3 ± 3.7^e^	—	—
MLE (*n* = 6)	—	2.3 ± 1.1	7.3 ± 4.1^e^	—	—
MMS (*n* = 6)	40.00	91 ± 3.6^c,d^	64.7 ± 4.2^e^	43.8 ± 5.1^e^	43 ± 9.5^f^
CP (*n* = 6)	25.00	45.8 ± 6.8^c^	31.3 ± 3.6^e^	18.3 ± 7.2^e^	11 ± 3.0^f^

		Damage frequency			
		DF ± SD	DF ± SD	DF ± SD	DF ± SD

Water (*n* = 6)	—	2.0 ± 1.0	7.2 ± 3.9^e^	—	—
MLE (*n* = 6)	—	2.3 ± 1.1	5.5 ± 2.4^e^	—	—
MMS (*n* = 6)	40.00	73.5 ± 1.9^c,d^	45.5 ± 5.7^e^	31.7 ± 3.6^e^	36.3 ± 10.7^f^
CP (*n* = 6)	25.00	34.8 ± 4.6^c^	22.3 ± 5.1^e^	14.5 ± 6.9^e^	10.2 ± 2.3^f^

DI: damage index; DF: damage frequency; *n*: number of individuals obtained from sum of independent experiments.

^a^Group sampled 24 h after treatment with an alkylating agent.

^b^Group sampled 48 h after treatment with an alkylating agent.

^c^Data significant in relation to water, EML in 24 h, *P* < 0.01 (ANOVA, Tukey).

^d^Data significant in relation to group CP, *P* < 0.01 (ANOVA, Tukey).

^e^Data significant in relation to 24 h at, *P* < 0.001 (*t*-test).

^f^Data significant in relation to 48 h at, *P* < 0.001 (*t*-test).
